# Nivolumab-associated acute glomerulonephritis: a case report and literature review

**DOI:** 10.1186/s12882-016-0408-2

**Published:** 2016-11-22

**Authors:** Kyungsuk Jung, Xu Zeng, Marijo Bilusic

**Affiliations:** 1 Department of Medicine, Fox Chase Cancer Center, 333 Cottman Ave, Philadelphia, PA 19111 USA; 2 Department of Pathology and Laboratory Medicine, Lewis Katz School of Medicine, Temple University, 3500N Broad St, Philadelphia, PA 19140 USA; 3 Department of Medical Oncology, Fox Chase Cancer Center, 333 Cottman Ave, Philadelphia, PA 19111 USA

**Keywords:** Immunotherapy, Nivolumab, Renal cell carcinoma, Acute kidney injury, Autoimmune nephritis, Case report

## Abstract

**Background:**

Immune checkpoint inhibitors are changing the landscape of oncology treatment as they are significantly improving treatment for multiple malignancies. Nivolumab, an anti-programmed death 1 antibody, is a US Food and Drug Administration-approved treatment for melanoma, non-small cell lung cancer, and kidney cancer but can result in a spectrum of autoimmune side effects. Adverse effects can occur within any organ system in the body including the colon, lung, liver, endocrine systems, or kidneys.

**Case presentation:**

A 70-year-old male with clear cell kidney cancer was admitted with acute kidney injury while on nivolumab. A kidney biopsy revealed diffuse tubular injury and immune complex-mediated glomerulonephritis. Electron microscopy of the specimen showed hump-like subepithelial deposits. Nivolumab was discontinued and the patient was started on a high dose of steroids. After 5 months of systemic corticosteroids and hemodialysis, the patient’s kidney function improved to his baseline level. Despite a prolonged interruption to treatment, immunosuppressive therapy did not compromise the anticancer effects of nivolumab.

**Conclusion:**

Immune-related adverse effects in the kidney can cause autoimmune glomerulonephritis as well as tubulointerstitial injury. In the literature, immune-related nephritis generally responded well to systemic corticosteroid treatment. Based on our experience, a prolonged course of a high dose of steroids and hemodialysis may be required to achieve an adequate treatment effect.

## Background

The field of oncologic immunotherapy is expanding rapidly. Since its introduction into clinical application for the treatment of melanoma [1, 2], immunotherapy has been studied in numerous trials for other types of cancer. Although treatments appear promising, immune checkpoint inhibition is associated with a unique category of side effects, termed immune-related adverse events (irAE) [3].

Programmed death 1 (PD1) is a transmembrane protein expressed on T cells, B cells, and natural killer cells. It binds to PD ligand 1 (PDL1) on the cell surface of tumor cells, inhibits cancer cell apoptosis, and down-regulates the functions of T cells [4, 5]. Nivolumab is a human immunoglobulin (Ig)G4 anti-PD1 monoclonal antibody, designed to augment an immunologic reaction against cancer cells. The medication is currently US Food and Drug Administration-approved for patients with advanced melanoma, non-small cell lung cancer, and renal cell carcinoma. irAE caused by nivolumab can affect any organ system including the lung, colon, liver, endocrine, kidney, skin, and brain. Grade 3 or 4 kidney injury was reported in 2% of the patients with renal cell carcinoma who were treated with nivolumab (creatinine >3 times above baseline or >4.0 mg/dL, or life-threatening consequences requiring dialysis) [6].

Kidney injury can cause diverse sequelae and potentially limit further oncologic treatment options, necessitating close follow-up and treatment. In clinical practice, irAE has been managed by treatment interruption and systemic corticosteroids as the first line, and tumor necrosis factor inhibitors or cytotoxic immunosuppressants as the second line [6].

In this report, we present a case of nivolumab-induced glomerulonephritis successfully treated with prolonged use of a high dose of steroids and hemodialysis.

## Case presentation

### History and initial presentation

The patient was a 70-year-old male with a past medical history of oxygen-dependent chronic pulmonary obstructive disease, squamous cell carcinoma of the right vocal cord (treated with definitive radiation therapy in November 1998), and stage 3b chronic kidney disease who was diagnosed with metastatic clear cell renal cell carcinoma in January 2013. Other pertinent past medical history included left renal vein thrombosis for which he was taking enoxaparin. The patient had a history of smoking (120 packs/year) but had quit smoking (120 packs/year) but had quit smoking in January 2013.

For the metastatic renal cell cancer, the patient was started on pazopanib 600 mg daily in February 2013, with a good initial response. However, medication was discontinued in December 2013 because of disease progression in the lungs and rib cage. He then began treatment with nivolumab 3 mg/kg every 2 weeks in December 2013. His disease initially responded well to the treatment. During the 10-month period while the patient was on nivolumab, left and right kidney tumors decreased by 19 and 13%, respectively, and adrenal masses decreased by 23% on both sides. He continued treatment until October 27, 2014 when he was found to have acute kidney injury (AKI), with a creatinine level of 10.08 mg/dL. His serum creatinine level the month prior was 1.67 mg/dL. He was admitted for evaluation and treatment for AKI. At the time of presentation, the patient had symptoms of generalized weakness, fatigue, and loss of appetite. His temperature was 35.7 °C (tympanic), and his blood pressure and heart rate were 135/70 mmHg and 79 beats/min, respectively. He showed a 1.7 kg weight gain over 1 month and there was the suggestion of 1+ bilateral ankle edema on physical examination. There was no flank pain or costovertebral angle tenderness.

### Hospital course

Upon admission, a metabolic panel revealed sodium 135 mmol/L, potassium 3.8 mmol/L, chloride 95 mmol/L, CO_2_ 28 mmol/L, total protein 6.1 g/dL, blood urea nitrogen (BUN) 58 mg/dL, and creatinine 10.08 mg/dL. Urinalysis was positive at >300 mg/dL for protein and 3+ for hemoglobin. On microscopic examination of urine, there were too-numerous-to-count red blood cells, 3–5 white blood cells, and 1–3 granular casts observed under high-power magnification. Fraction excretion of sodium was 2.2%. Serum C3 and C4 levels were within normal ranges. Hepatitis B surface antigen, hepatitis C antibody, anti-nuclear antibody, anti-double strand DNA antibody, glomerular basement membrane antibody, cytoplasmic anti-neutrophil cytoplasmic antibody, and perinuclear anti-neutrophil cytoplasmic antibody were all negative. Ultrasound revealed solid masses in the interpolar region of the right kidney and upper pole of the left kidney, representative of his known renal cell carcinoma. Otherwise, kidney sizes were within the normal range and there was no evidence of hydronephrosis.

**Fig. 1:**
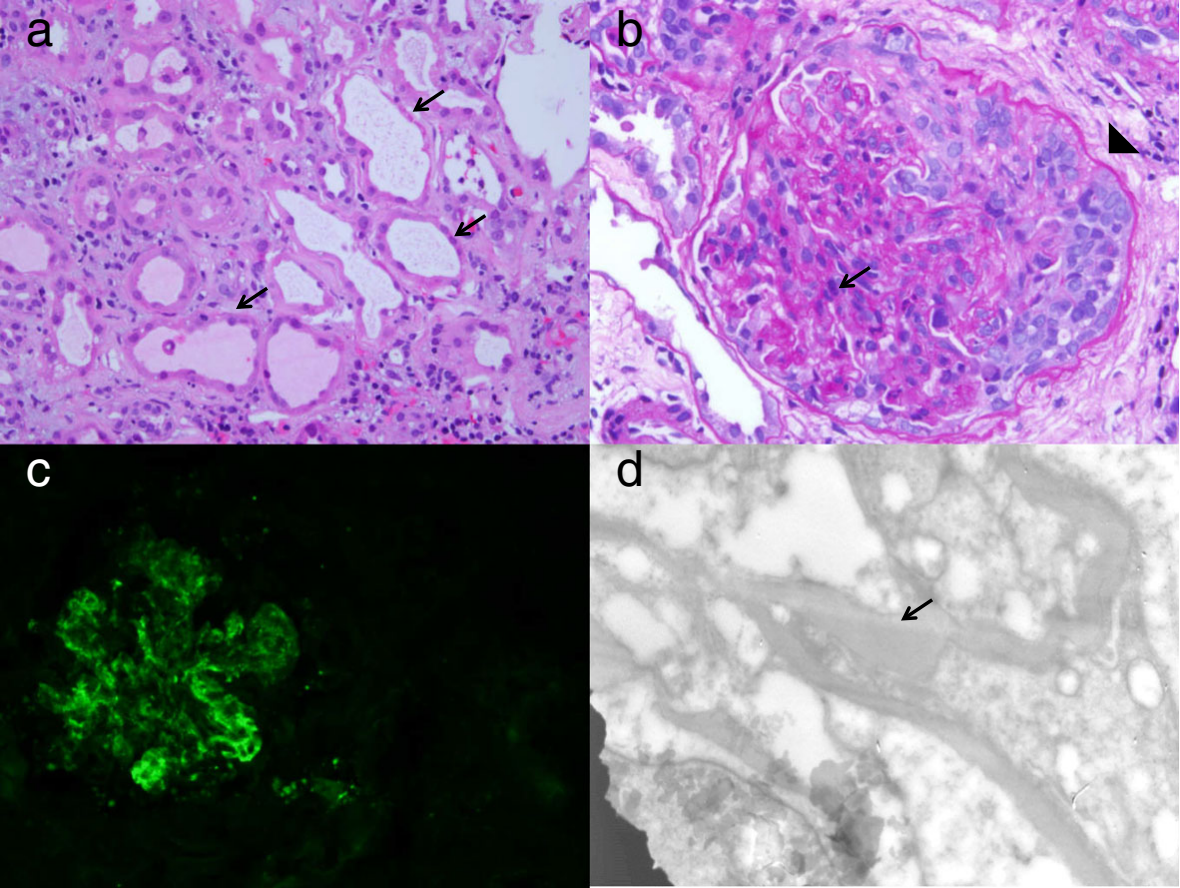
Microscopic examinations of kidney biopsy specimens. **a** and **b** Hematoxylin and eosin staining of the kidney biopsy specimen revealed interstitial infiltrate with tubular injury (*arrows*), glomerulonephritis with cellular crescents (*arrowhead*), and mesangial proliferation (*arrow*). **c** Immunofluorescence staining for IgA deposits. **d** Electron microscopic picture of subepithelial deposit (*arrow*)

Biopsy of the right lower pole of the kidney was performed on October 29, 2014. Light microscopic examination demonstrated diffuse tubular injury with vacuoles and immune complex-mediated glomerulonephritis with cellular crescents and necrosis. There was moderate interstitial inflammation with lymphocytes observed. With immunofluorescence, there was diffuse granular mesangial staining for IgA, C3, and kappa and lambda light chains. The specimen was also sent for electron microscopic examination. One glomerulus with severe cellular crescents was selected for examination and demonstrated several hump-like subepithelial deposits and no subendothelial deposits. There was partial podocyte foot process effacement. Proximal tubules were flattened with simplified tubular epithelium and shorter microvilli. Pathologic examinations confirmed the final diagnosis of acute toxic-type tubular injury and IgA-dominant acute post-infectious glomerulonephritis. Pictures of microscopic examinations are shown in Fig. 1.

Although there was the possibility of post-infectious glomerulonephritis based on findings of glomerular deposits in the kidney biopsy, the patient did not show symptoms of streptococcal infection such as pharyngitis or a rash prior to admission. The patient’s C3 level was normal and he was not hypertensive. Based on patient’s previous exposure to nivolumab and lymphocyte infiltration observed in the biopsy, immunotherapy-induced kidney injury was taken into consideration. Nivolumab was discontinued and methylprednisolone administration 40 mg intravenously twice a day was started. The following day, serum potassium increased to 5.6 mmol/L, and creatinine and BUN were elevated at 11.01 and 63 mg/dL, respectively. Nephrology was consulted and hemodialysis was initiated. Methylprednisolone was increased to 40 mg three times a day (1 mg/kg/day). After the biopsy report, the patient was started on pulse dose steroids, methylprednisolone 1 g intravenously daily for 3 days, followed by methylprednisolone 40 mg intravenously three times a day. The creatinine level decreased after steroid treatment and hemodialysis. Four days later, steroids were changed to oral prednisone 40 mg twice a day and the patient was discharged on steroid treatment and outpatient hemodialysis. On the day of discharge, his creatinine level was 8.80 mg/dL.

### Follow-up and outcome

**Fig. 2:**
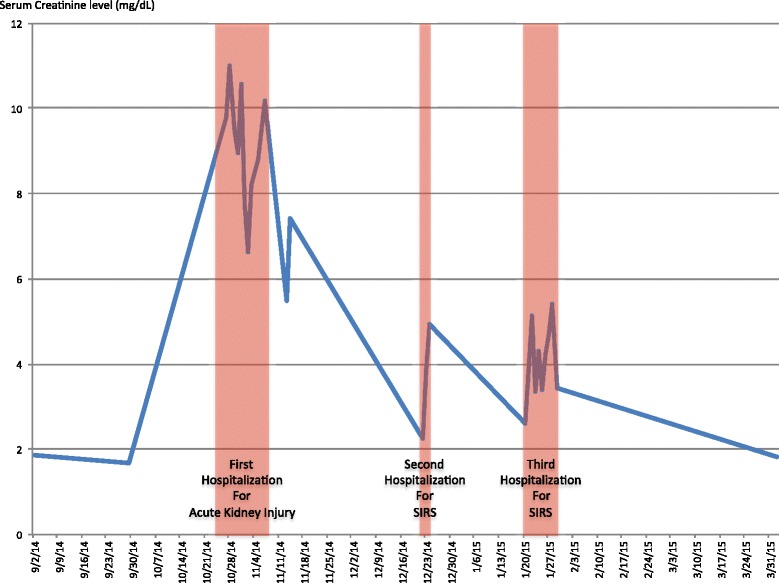
Serum creatinine changes over the 6-month treatment period

One month after discharge, the patient was admitted with a fever, rash, tachycardia, and leukocytosis, consistent with systemic inflammatory response syndrome (SIRS). The source of infection was unclear as blood and urine cultures were negative. The patient had generalized patchy skin lesions with desquamation, most prominent at the bilateral proximal arms and upper torso. Biopsy of skin lesions was deferred, as they were thought to be an irAE and already clinically improving with steroid treatment. The patient was discharged after a short course of intravenous antibiotics. The dose of prednisone was increased at this time. Another month after the second hospitalization, the patient was re-admitted with fever, tachycardia, and hypotension. Again, there was no compelling source of infection identified after an extensive diagnostic work up. During the third hospitalization of 9 days, he received a stress dose of hydrocortisone 100 mg three times a day. Upon discharge, he resumed a tapering course of steroids, starting with prednisone 60 mg daily. The patient tolerated the prolonged course of oral steroids well with no apparent adverse effects. Oral prednisone was stopped at the end of February 2015. In April 2015, his serum creatinine level was 1.81 mg/dL and BUN was 13 mg/dL. Hemodialysis was discontinued on April 27, 2015. The last contact with the patient was on March 30, 2016 and his kidney function remained stable at the time. Change in serum creatinine over the 6-month treatment period is shown in Fig. 2.

While recovering from nivolumab toxicity, the patient did not receive any treatment for renal cell carcinoma. In spite of the prolonged systemic corticosteroid treatment, anti-tumor activity seemed to continue as the tumors in bilateral kidneys and adrenal glands decreased in sizes for 18 months (left adrenal gland: 3.7 to 2.4 cm, right adrenal gland: 7.0 to 5.8 cm, left kidney mass: 6.0 to 3.4 cm, right kidney mass: 7.0 to 5.8 cm; all measurements in longest diameter). In March 2016, he was started on axitinib 3 mg twice a day for symptomatic disease progression (worsening of rib lesion).

## Conclusions

irAE are encountered more frequently in daily oncologic practice with the increased use of immune checkpoint inhibitors. Immune checkpoint inhibition involves two major transmembrane proteins, cytotoxic T-lymphocyte antigen 4 and PD1. Nivolumab, a monoclonal anti-PD1 antibody, blocks T cell inhibition and stimulates the immunologic response towards cancer cells, but it may also impair the self-tolerance of the immune system. This potential side effect can occur in any organ of the body but is known to predominately occur in the gastrointestinal tract, lung, liver, and endocrine system [7].

In clinical trials of nivolumab or other anti-PD1 antibodies, the renal system was not affected as frequently as other organ systems. In a population of patients with non-small cell lung cancer, seven of 287 patients (2.4%) who were treated with nivolumab developed renal injury, but all events were either grade 1 or 2 [8]. Among patients with melanoma treated with pembrolizumab (anti-PD1 antibody), one of 277 patients (0.4%) experienced nephritis with renal failure [9]. Another anti-PD1 antibody, lambrolizumab, was associated with renal failure in three of 135 patients (2%) with melanoma, with two of these three patients having grade 3 or 4 adverse events [10].

Table 1 summarizes case reports on irAE affecting the kidney, identified in a review of the literature. Fadel et al. reported a case of lupus nephritis that occurred after use of ipilimumab and was resolved after steroid treatment [11]. Six cases of ipilimumab-associated nephritis were reported [12–15]. The articles generally reported successful treatment of ipilimumab-associated nephritis using systemic steroids. In addition, there were three cases of AKI caused by anti-PD1 antibody, such as nivolumab or pembrolizumab. Two patients who had nivolumab-related kidney injury were successfully treated with steroids [16], and one patient who developed pembrolizumab-related kidney injury showed improvement of nephritis after steroid treatment [17].

**Table 1:** Reports in the literature of immunotherapy-associated renal adverse events and treatment

Author	No. of patients	Age	Gender	Medication	Cancer	Kidney biopsy	Treatment	Outcome
Fadel et al. [11] 2009	1	64	M	Ipilimumab	Melanoma	Extra-membranous and mesangial deposits of immunoglobulin (consistent with lupus nephritis)	Steroids	Resolved
Forde et al. [12] 2012	1	59	M	Ipilimumab	Melanoma	Not available	Steroids; no dialysis	Resolved
Voskens et al. [13] 2013	1	53	F	Ipilimumab	Mucosal	Not available	Steroids	Resolved
	2	72	F	Ipilimumab	Unknown primary	Not available	Steroids	Resolved
Izzedine et al. [14] 2014	1	72	M	Ipilimumab	Melanoma	Interstitial inflammation and poly-nuclear infiltration in glomerulus	Steroids	Resolved
	2	60	F	Ipilimumab	Melanoma	Tubulointerstitial inflammation with necrosis and two non-necrotizing granulomas	Steroids	Resolved
Thajudeen et al. [15] 2015	1	74	M	Ipilimumab	Melanoma	Interstitial edema with infiltrate of lymphocytes and granulomas	Steroids	Resolved; ipilimumab resumed
Vandiver et al. [16] 2016	1	58	F	Nivolumab	Melanoma	Not available	Steroids	Resolved
Hofmann et al. [17] 2016	1	52	M	Nivolumab	Melanoma	Not available	Steroids and normal saline	Resolved; nivolumab resumed
	2	73	M	Pembrolizumab	Melanoma	Not available	Steroids	Improved

Although alterations in immunologic self-tolerance theoretically account for renal dysfunction, specific mechanisms among existing case reports are diverse. Biopsy results also varied. One patient was diagnosed with lupus nephritis during treatment with ipilimumab [11]. Diagnosis was made based on the patient being positive for anti-nuclear and anti-double-stranded DNA antibodies. Electron microscopy confirmed the presence of granular, electron-dense extra-membranous deposits. Izzedine et al. reported two cases of acute interstitial nephritis associated with ipilimumab [14]. In a more recent case, reported by Thajudeen et al., a biopsy revealed granulomas and interstitial infiltration with lymphocytes, eosinophils, and plasma cells [15]. In the current case, the biopsy revealed acute tubular injury and immune complex-mediated glomerulonephritis.

Regardless of the etiology or biopsy results, patient’s kidney functions generally improved after steroid treatment. We found no case in our review of the literature in which tumor necrosis factor inhibitors or cytotoxic immunosuppressants were required. In some reviewed cases, immunotherapy resumed after kidney function improved. In the current case, nivolumab was permanently discontinued and the patient continued on a lengthy tapering course of steroids for approximately 4 months. Even after prolonged use of systemic corticosteroids and discontinuation of nivolumab, the immunologic anti-tumor effect appeared to persist and the tumors in his kidneys shrank in size. In the literature, there was a case of a melanoma patient who experienced continued tumor regression in spite of discontinuation of ipilimumab and daily administration of systemic corticosteroids for irAE [18]. In another case of a patient with melanoma treated with ipilimumab, both steroids and infliximab were used for grade 3 colitis, and there was no disease progression after approximately 3 years [19]. In a clinical trial, corticosteroids for treatment of irAE did not impact the clinical activity of ipilimumab in advanced melanoma patients [20].

In the current case, the possibility of post-infectious glomerulonephritis was raised based on glomerular deposits in the kidney biopsy. However, the patient did not have symptoms of pharyngitis or a skin infection prior to admission. Lack of hypertension and a normal C3 level were also not consistent with post-infectious glomerulonephritis. Autoimmune glomerular injury was determined as a major component of the etiology in our case, based on the temporal relationship between the use of nivolumab and the onset of AKI. To the best of the authors’ knowledge, this is the first case of nivolumab-associated glomerulonephritis confirmed by light and electron microscopic findings.

The patient in our case was twice admitted with unexplained SIRS. Both hospitalizations occurred while on a decreasing dose of prednisone. After extensive diagnostic testing for infection, there was no clear source of SIRS. Pyrexia has been reported as a side effect of immunotherapy in clinical trials, but our patient had severe SIRS with hypotension requiring admission to the intensive care unit. Although the potential relationship between immunotherapy and SIRS is intriguing, more evidence is required before a causal link can be established.

In summary, use of systemic corticosteroids has been generally successful in achieving an optimal treatment response for immunotherapy-associated renal dysfunction. Nivolumab manufacturer recommends 0.5–1 mg/kg/day prednisone equivalents for grade 2 or 3 renal dysfunction, and if no improvement occurs, 1–2 mg/kg/day prednisone equivalents and discontinuation of nivolumab. For life-threatening grade 4 renal dysfunction, the recommendation is to start with 1–2 mg/kg/day prednisone equivalents and permanently discontinue nivolumab [6]. Based on our experience, a pulse dose of steroids can be used for resistant renal dysfunction. Although treatment responses may not be ostensible initially, a presumptive decision of treatment failure should be avoided. As in our case, patients may require a prolonged course of systemic corticosteroids and hemodialysis, and kidney function may improve months later.

## Abbreviations

AKI: Acute kidney injury
irAE: Immune-related adverse events
PD1: Programmed death 1
PDL1: PD ligand 1
SIRS: Systemic inflammatory response syndrome
